# Transplantation of gut microbiota from old mice into young healthy mice reduces lean mass but not bone mass

**DOI:** 10.1080/19490976.2023.2236755

**Published:** 2023-07-20

**Authors:** Lina Lawenius, Carrie Cowardin, Louise Grahnemo, Julia M. Scheffler, Karin Horkeby, Cecilia Engdahl, Jianyao Wu, Liesbeth Vandenput, Antti Koskela, Juha Tukkanen, Eivind Coward, Kristian Hveem, Arnulf Langhammer, Sanna Abrahamsson, Jeffrey I. Gordon, Klara Sjögren, Claes Ohlsson

**Affiliations:** a Department of Internal Medicine and Clinical Nutrition, Institute of Medicine, Sahlgrenska Osteoporosis Centre, Centre for Bone and Arthritis Research at the Sahlgrenska Academy, University of Gothenburg, Gothenburg, Sweden; b The Edison Family Center for Genome Sciences and Systems Biology, Washington University in St. Louis, St. Louis, MO, USA; c Department of Rheumatology and Inflammation Research, Institute of Medicine, Sahlgrenska Osteoporosis Centre, Centre for Bone and Arthritis Research at the Sahlgrenska Academy, Sahlgrenska Academy University of Gothenburg, Gothenburg, Sweden; d Department of Anatomy and Cell Biology, Faculty of Medicine, Institute of Cancer Research and Translational Medicine, University of Oulu, Oulu, Finland; e K.G. Jebsen Center for Genetic Epidemiology, Department of Public Health and Nursing, NTNU, Norwegian University of Science and Technology, Trondheim, Norway; f HUNT Research Centre, Department of Public Health and Nursing, Norwegian University of Science and Technology, Levanger, Norway; g Bioinformatics and Data Centre, Sahlgrenska Academy, University of Gothenburg, Gothenburg, Sweden; h Department of Drug Treatment, Region Västra Götaland, Sahlgrenska University Hospital, Gothenburg, Sweden

**Keywords:** Gut microbiota, germ-free mice, gut microbiota transplantation, bone mass, osteoporosis, lean mass, sarcopenia, bacteroides ovatus, HUNT cohort

## Abstract

Aging is associated with low bone and lean mass as well as alterations in the gut microbiota (GM). In this study, we determined whether the reduced bone mass and relative lean mass observed in old mice could be transferred to healthy young mice by GM transplantation (GMT). GM from old (21-month-old) and young adult (5-month-old) donors was used to colonize germ-free (GF) mice in three separate studies involving still growing 5- or 11-week-old recipients and 17-week-old recipients with minimal bone growth. The GM of the recipient mice was similar to that of the donors, demonstrating successful GMT. GM from old mice did not have statistically significant effects on bone mass or bone strength, but significantly reduced the lean mass percentage of still growing recipient mice when compared with recipients of GM from young adult mice. The levels of propionate in the cecum of mice receiving old donor GM were significantly lower than those in mice receiving young adult donor GM. *Bacteroides ovatus* was enriched in the microbiota of recipient mice harboring GM from young adult donors. The presence of *B. ovatus* was not only significantly associated with high lean mass percentage in mice, but also with lean mass adjusted for fat mass in the large human HUNT cohort. In conclusion, GM from old mice reduces lean mass percentage but not bone mass in young, healthy, still growing recipient mice. Future studies are warranted to determine whether GM from young mice improves the musculoskeletal phenotype of frail elderly recipient mice.

## Introduction

Aging is associated with sarcopenia and osteoporosis, leading to high economic and social burden.^1,2^ Sarcopenia, an age-related loss of muscle mass and function, is associated with falls, functional decline, frailty, fractures, and mortality.^3^ The human gut microbiota (GM), whose acquisition begins at birth, is considered a multicellular organ that communicates with and affects its host.^4^ The composition of GM is modulated by environmental factors, such as diet and antibiotic treatment.^5–7^ Major shifts occur as the GM is assembled during the first several years of postnatal life and as a manifestation of senescence in the elderly.^8^ The reduced diversity of GM in elderly individuals has been associated with both frailty and several different diseases.^9,10^ However, it is unknown if age-related changes in the GM cause physical changes associated with aging, such as fragile bones and low lean mass percentage.

Bone mass in mice peaks at around 5 months of age^11^ while absolute lean mass continues to increase throughout life. However, since the body weight increases more, the lean mass percentage decreases with age, with lean mass percentage peaking at around 5 months of age.^12^ CD4+ lymphocytes and regulatory T (Treg) cells in the bone marrow are important for bone metabolism and have been reported to change with age. Colonization of germ-free (GF) mice results in an acute loss of bone associated with an increased frequency of CD4+ lymphocytes in bone marrow and Treg cells are possible mediators of gut microbiota effects on bone.^13–15^ In both mice and humans Treg cells increase with age.^16,17^

The GM has been implicated as a regulator of bone mass.^13,18,19^ Members of the GM produce short-chain fatty acids (SCFA) such as acetate, propionate, and butyrate by fermenting prebiotic fibers.^20^ Studies have shown that SCFAs can protect from bone loss by inhibiting bone-degrading osteoclasts^21^ and by inducing bone-forming osteoblasts.^15^ Several studies have reported that probiotics, such as *Lactobacillus, Bacillus*, and *Bifidobacterium* protect from bone loss in estrogen-deficient rodents.^22–27^ In humans, treatment with certain strains of *Lactobacillus* protects against tibial bone loss in old women^28^ and against lumbar spine bone loss in postmenopausal women.^29^

The GM can also regulate muscle mass. Compared with conventionally raised mice, germ-free (GF) mice have lower skeletal muscle mass, which can be restored by GM transplantation.^30,31^ Furthermore, antibiotic-treated mice showed reduced muscle mass.^32–34^ Treatment with SCFAs increases muscle weight in GF mice^30^ and protects from muscle loss during aging in conventionally raised mice.^35^ Treatment with probiotics protects old mice and age-accelerated mice from muscle loss^36,37^and can improve the relative muscle weight in young adult mice.^38^ In humans, the effect of probiotics on lean mass, an estimate of muscle mass, is poorly studied. In a small study (*n* = 54), probiotic treatment for 6 weeks improved lean mass.^39^

GF mice lack all microorganisms, which makes them a suitable model for analyzing the effect of GM on the skeleton and relative lean mass.^40^ The current study aimed to determine if age-related changes in GM composition contribute to the reduced bone mass and relative lean mass observed in old mice compared to young adult mice. Therefore, we colonized GF recipient mice with GM from old and young adult donor mice to investigate whether low bone mass and/or relative lean mass phenotypes are transmissible from old mice to young healthy GF recipient mice.

## Results

### Old donor mice have lower lean mass percentage and bone mass and altered GM composition compared with young adult donor mice

**Figure 1.**
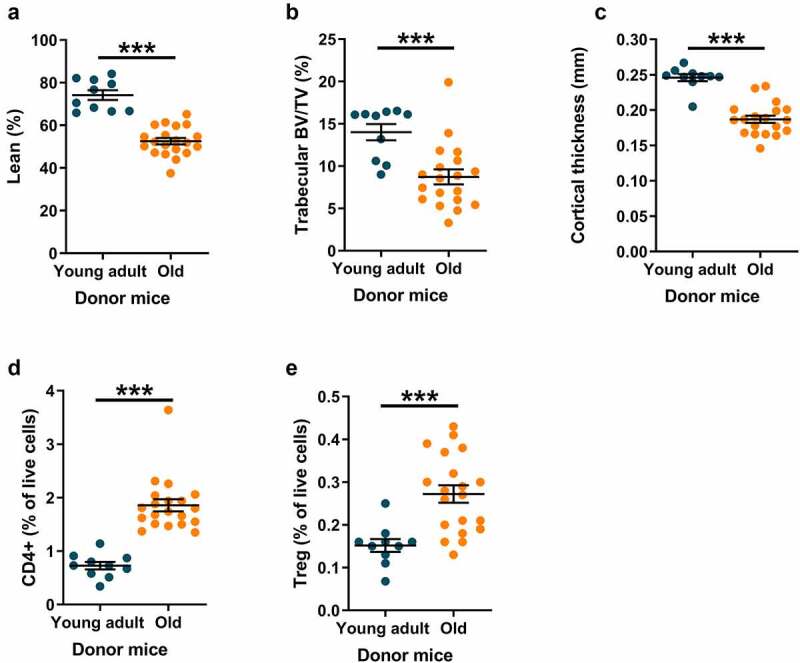
Old donor mice have lower lean mass and bone mass compared with young adult donor mice.

**Figure 2.**
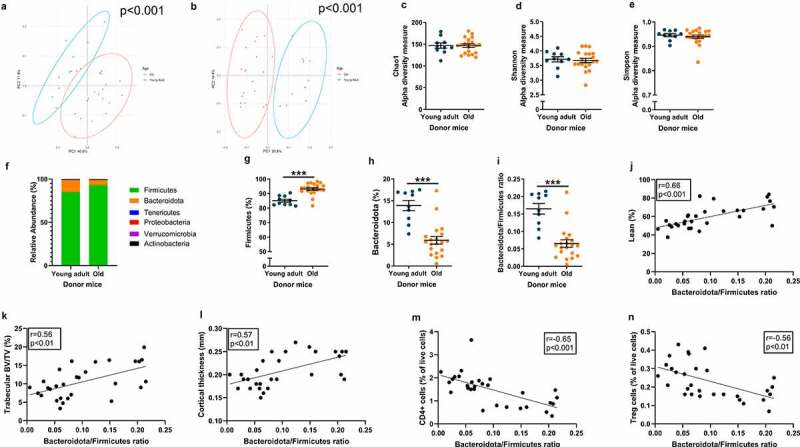
Old donor mice have altered GM composition compared with young adult donor mice.

We started by characterizing 21-month-old (old) and 5-month-old (young adult) female C57BL/6J mice that were used as donors for gut microbiota transplantation (GMT) studies. DXA analyses revealed that old mice had a statistically significantly lower lean mass percentage than young adult mice (Figure 1a). Old mice showed statistically significant decreases in the tibial trabecular bone volume fraction (BV/TV in the proximal metaphyseal region, Figure 1b) and cortical bone mass (cortical thickness in the mid-diaphyseal region, Figure 1c) compared with young adult mice. The percentage of CD4+ T lymphocytes and Treg cells was significantly higher in the bone marrow of old mice than in that of young adult mice (Figure 1d,e, Fig. S1-S3). We analyzed bacterial membership in cecal contents by 16S rRNA amplicon sequencing followed by principal coordinate analysis (PCoA) and found that the gut microbial communities of old and young adult donors had statistically significant differences in beta-diversity, whether quantified by weighted or unweighted UniFrac distances (Figure 2a,b). In contrast, there were no significant differences in the alpha diversity of cecal communities between old and young adult mice, as measured by Chao1, Shannon, or Simpson indices (Figure 2c–e).

21-month-old mice (old) and 5-month-old mice (young adult) were analyzed by dual-energy x-ray absorptiometry (DXA) to determine lean% (a). The tibia was dissected from each animal and analyzed by high-resolution microCT (µCT) to determine the trabecular BV/TV (b) and cortical thickness (c). Bone marrow cells were harvested from the femur and stained for CD4, Foxp3, and CD25 to analyze CD4+ cells (d) and regulatory T cells (Treg, CD4+CD25+Foxp3+, E). Values are given as mean ± SEM. Data were statistically analyzed using Student’s *t-*test. ***p ≤ 0.001.

The cecal microbial communities of conventionally raised 21-month-old mice (old) and 5-month-old mice (young adult) were analyzed by bacterial V4-16S rRNA amplicon sequencing and principal coordinate analysis (PCoA) based on weighted (a), and unweighted (b) UniFrac distances. Alpha diversity was determined by Chao1 (c), Shannon (d), and Simpson (e). The relative abundance of all phyla (f), Firmicutes (g), Bacteroidota (h), and Bacteroidota/Firmicutes ratio (i) were analyzed. The correlation of Bacteroidota/Firmicutes ratio vs % lean body mass (j), trabecular BV/TV (k), cortical thickness (l), CD4+ cells (m), and Treg cells (n) were analyzed. Data in panels A and B were statistically analyzed by permutational multivariate analysis of variance (PERMANOVA), in panels C-E and G-I by Student’s *t-*test, and in panels J-N by Pearson’s correlation test. ***p ≤ 0.001. Values are given as the mean ± SEM.

Analyses of the GM composition at the phylum level revealed clear differences between old and young adult donor mice (Figure 2f), including an increase in the relative abundance of Firmicutes and a decrease in the relative abundance of Bacteroidota and the Bacteroidota/Firmicutes ratio in old mice compared with young adult mice (Figure 2g–i). The Bacteroidota/Firmicutes ratio was significantly positively associated with lean mass percentage (Figure 2j) and bone mass (Figure 2k, l), while it was significantly negatively associated with the frequency of bone marrow CD4+ T lymphocytes (Figure 2m) and Treg cells (Figure 2n).

### The GM is transferred to recipient mice

**Figure 3.**
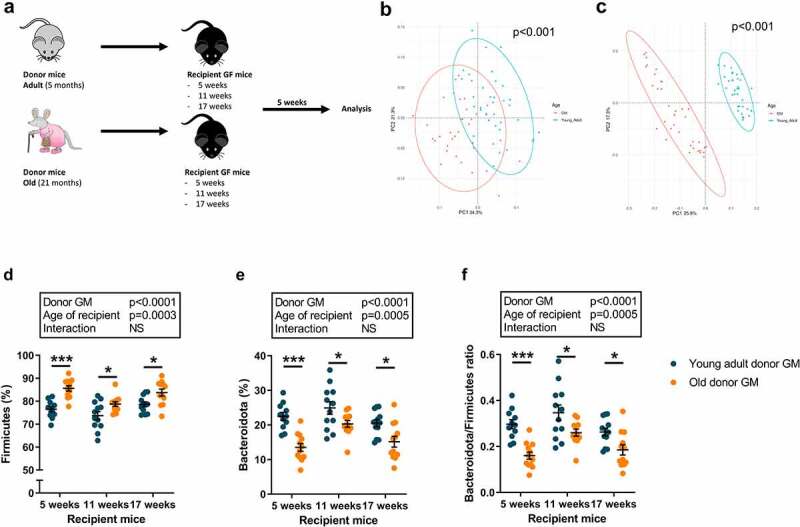
GM transplantation to recipient GF mice.

Old or young adult GM was used to colonize young healthy GF mice in three independent GMT experiments using still growing recipient mice at 5 (Study 1) or 11 (Study 2) weeks of age, or recipient mice at 17 weeks of age when bone growth is minimal (Study 3; Figure 3a). PCoA of weighted and unweighted UniFrac distances revealed that after 5 weeks of colonization, the bacterial composition of the cecal microbial communities of recipient mice colonized with GM from old and young adult mice differed from one another in a statistically significant fashion (Figure 3b,c and Fig. S4). The communities of recipient animals were similar to the original donors when evaluating donor mice in all three studies together and when evaluating each individual study separately (Fig. S5). Taken together, these analyses provide evidence of a successful GMT.

Cecal microbiota from old or young adult donor mice was transplanted to GF mice of different ages. Study protocol (a). The cecal microbial communities of recipient mice were analyzed by bacterial V4-16S rRNA amplicon sequencing and principal coordinate analysis (PCoA) based on weighted (b) and unweighted (c) UniFrac distances. The relative abundances of Firmicutes (d) and Bacteroidota (e), and the Bacteroidota/Firmicutes ratio (f) were determined. Data in panels B and C were statistically analyzed by permutation multivariate of variance (PERMANOVA), and in panels D-F were statistically analyzed for the overall effect of age of donor mice (old vs young adult), age of recipient mice (GF mice), and their interaction were calculated using two-way ANOVA followed by Šídák post hoc test to correct for multiple comparisons. ***p ≤ 0.001, **p ≤ 0.01, and *p ≤ 0.05.

### The GM from old mice reduces lean mass percentage but not bone mass in young healthy still growing recipient mice compared with GM from young adult donors

Analyses at the phyla level revealed clear differences between mice colonized with GM from old donors and young adult donors (Fig. S6A-C). In line with the data for the donor mice, the cecal microbiota of mice that had been colonized with GM from old donors had significantly higher levels of Firmicutes, lower levels of Bacteroidota, and a lower ratio of Bacteroidota/Firmicutes compared with mice that had been colonized with GM from young adult donors (Figure 3d–f). Moreover, both bacterial richness and evenness were significantly lower in recipient mice colonized with GM from old compared to young adult donors, as defined by Chao1, Shannon, and Simpson indices (Fig. S6D-F). In addition, the levels of SCFA propionate and succinate, an intermediate in SCFA synthesis, in cecal contents were significantly lower in recipient mice colonized with old GM than in young adult GM, as analyzed by two-way ANOVA (Fig. S7).

To investigate if the transplantation of GM induced an inflammatory response that differed between young adult and old GM, we measured serum amyloid A (SAA) in serum 35 days after colonization. We found no difference in serum SAA levels between mice receiving GM from old compared to young adult mice (Fig S8).

**Figure 4.**
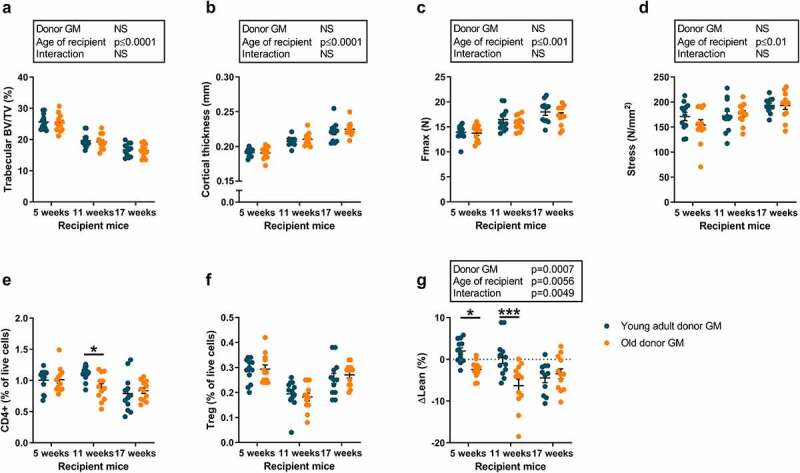
The GM from old mice reduces relative lean mass but not bone mass in young healthy still growing recipient mice compared with the GM from young adult donors.

Next, we determined whether the observed differences in bone mass, bone marrow immune phenotypes, and lean mass percentage between old and young adult mice could be transferred from old donors to young healthy recipients. GM from old donors did not have statistically significant effects on trabecular bone (Figure 4a), cortical bone (Figure 4b), bone strength parameters (Figure 4c, d), or bone marrow immune phenotypes (Figure 4e,f, Fig. S1-S3) in still growing (5-week-old and 11-week-old) or non-growing adult (17-week-old) recipient mice. However, GM from old compared to young mice affected the lean mass percentage of recipient mice (Figure 4g). The effect was modulated by the age of the recipient mice; post hoc analysis revealed that a significant effect was observed in the young still growing (5-week-old and 11-week-old) recipient mice, but not in the non-growing adult recipients (17-week-old). GM from old compared to young mice affected the total body lean mass but not the total body fat mass (Fig. S9). The age of the donor mice did not significantly affect the body weight or the relative weights of the dissected tissues (Fig. S10).

Cecal microbiota from old or young adult donor mice was transplanted to GF mice of different ages. At the end of the experiment, tibia and humerus were dissected. The tibia were analyzed by high-resolution microCT (µCT) to determine trabecular BV/TV (a) and cortical thickness (b). The humerus was analyzed by three-point bending to measure maximum force (Fmax, c) and the bone quality parameter material stress was calculated (d). Bone marrow cells were harvested from femur and stained for CD4, Foxp3, and CD25 to analyze CD4+ cells (e) and regulatory T cells (Treg, CD4+CD25+Foxp3+, panel f). Body composition was determined at the time of gavage and 5 weeks later using qMR to calculate difference in lean mass percentage (ΔLean%, g). Values are given as the mean ± SEM. Data in panels A-D and G were analyzed by two-way ANOVA to analyze the overall effect of age of donor mice (old vs young adult), age of recipient GF mice, and their interaction followed by Šídák post hoc test to correct for multiple comparisons. Data in panels E and F were analyzed by Student’s *t-*test followed by Bonferroni correction since samples from the three sub-studies were analyzed on different occasions and cannot be compared due to interassay variation. ***p ≤ 0.001, **p ≤ 0.01, and *p ≤ 0.05.

### Presence of *B.* *ovatus* in cecum/feces is a marker of high relative lean mass in both mice and humans

LEfSe analyses revealed that *Bacteroides ovatus* was the only taxa analyzed at the species level that was enriched in recipient mice colonized with GM from young adult donors compared with old donors (Fig. S11). The relative abundance of *B. ovatus* was directly associated with the change in lean mass percentage in still growing recipient mice (Spearman correlation: 5-week-old recipients, *r* = 0.62, *p* = .0016; 11-week-old recipients, *r* = 0.64, *p* = .0007, Table 1). In addition, *B. ovatus* was not present in cecal samples from any of the old donor mice having low lean mass percentage (*n* = 20), while it was present at a relative abundance of 1.8 ± 0.3% (mean ± SEM) in cecal samples from all young adult donor mice (*n* = 10), having high lean mass percentage.

**Table 1.** Correlation between relative abundance of *B. ovatus* and change in percentage of lean mass in mice.

Age of recipient mice	r-value	p-value
5 weeks	0.62	p=.0016
11 weeks	0.64	p=.0007
17 weeks	−0.08	NS

5-, 11- and 17-week-old GF mice received microbiota transplants from young adult or old donors and were euthanized 5 weeks later (*n* = 12 mice/treatment group). The percentage of lean body mass was determined at the beginning and at the end of the experiment by qMR. At the end of the experiment, cecal contents were collected, the PCR products of variable region 4 of bacterial 16S rRNA genes were sequenced and the relative abundances of amplicon sequence variants (ASVs) were defined. Data were analyzed by Spearman correlation to determine the correlation coefficient (r-value) of the relative abundance of ASVs assigned to *B. ovatus* and Δlean%.

We next determined whether the presence of *B. ovatus* was associated with lean mass adjusted for fat mass in the large human population-based Norwegian HUNT4 study (*N* = 4,966). *B. ovatus* was present in the fecal samples of 79.6% of the subjects in the cohort based on a quantitative PCR assay. The presence of *B. ovatus* was positively associated with both total body lean mass (Beta 0.29, SE 0.14, expressed as kg lean mass for the presence of *B. ovatus*, *p* = .033) and appendicular lean mass (Beta 0.14, SE 0.07, *p* = .031; models adjusted for age, sex, height, body fat mass, batch, and batch-specific covariates). Thus, the presence of *B. ovatus* is associated with high relative lean mass in both mice and humans.

## Discussion

In the present study, we showed that old mice have reduced relative lean and bone mass and altered GM composition compared with young adult mice. GM from old and young adult mice were transplanted into GF mice in three separate experiments. At the end of each experiment, the recipient mice had a similar GM to their donor mice, confirming successful GMT. GM from old mice did not affect bone mass, but reduced relative lean mass in still growing recipient mice compared with GM from young adult mice. *B. ovatus* was enriched in recipient mice colonized with GM from young adult donor mice and we found that *B. ovatus* was positively associated with lean mass in mice. Using a large human cohort, we observed that the presence of *B. ovatus* was positively associated with lean mass, adjusted for fat mass in humans.

Bone mass^11,41^and relative lean mass^12^ are reduced with age in mice. We found similar results in the donor mice of the present study, in which old donor mice had lower bone mass and lean mass percentage than young adult donor mice. Old donor mice had increased levels of CD4+ lymphocytes and Treg cells in the bone marrow compared to young adult donors. This finding is in line with earlier studies demonstrating that CD4+ lymphocytes are negatively associated with bone mass in mice^13^ and that Treg cells are increased in aged mice.^17^

Previous studies have demonstrated that GM composition differs between young adults and old mice.^42–44^ At the phyla level, old donor mice had a higher relative abundance of Firmicutes and a lower relative abundance of Bacteroidota compared to young adult donors, resulting in a lower Bacteroidota/Firmicutes ratio in old donor mice compared with young adult donor mice in the present study. This finding confirms previous studies demonstrating that the relative abundance of Firmicutes increases and that of Bacteroidota decreased with increasing age in mice.^45^ In addition, we observed positive associations between Bacteroidota/Firmicutes ratio, bone mass, and relative lean mass in donor mice. Therefore, we hypothesized that age-related changes in GM could be linked to reduced bone mass and relative lean mass in old mice.

To test this hypothesis, we colonized young healthy GF mice using still growing recipient mice (5-week-old and 11-week-old), or recipient mice with minimal bone growth at 17 weeks of age, with GM from young adult or old donor mice. The gut communities of the recipient mice were similar to those of the donors, and these findings verified successful GMT.

Previous studies have demonstrated that GM can regulate skeletal homeostasis.^13,18,19^ In the present study, GM from old donor mice did not affect the bone mass or bone strength in recipient mice. In an earlier study, Wang *et al*. showed that multiple treatments with fecal samples from old conventionally raised rats three times per week for 24 weeks (a total of 72 treatments) reduced trabecular bone mass in conventionally raised young rats, but control rats were not given fecal samples from young rats. Thus, that study was not a comparison of the effect of GM derived from donors of different ages, and the observed effect may very well be the result of non-GM related factors present in the feces, delivered repeatedly 72 times over the course of 24 weeks.^46^ In our study, GM from old donors did not significantly reduce bone mass in young, healthy gnotobiotic mouse recipients. However, we cannot exclude the possibility that GM from young adult donor mice can improve bone mass in frail old mice.

Old donor mice had higher levels of Treg cells and CD4+ lymphocytes than young adult donors. However, the levels of Treg cells were not affected by colonizing recipient mice with GM from old donors compared to young adult donors, and levels of CD4+ lymphocytes were not affected in two of the three mice experiments, demonstrating that the observed bone marrow lymphocyte phenotypes in old donor mice are generally not transferred to recipient mice through GMT.

Recipient mice colonized with GM from old donor mice had reduced relative lean mass compared to recipient mice colonized with GM from young adult donors. Interestingly, this effect on lean mass percentage was modulated by the age of the recipient mice, and a significant effect was observed in young still growing recipient mice (5 and 11 weeks of age) but not in young adult recipient mice with slow growth (17 weeks of age). Thus, the low lean mass percentage in old mice can be transferred to young adult recipient mice through GMT. Further studies are needed to evaluate whether GM from young adult mice to old frail recipient mice can improve the relative lean mass.

In the present study, SCFAs were increased in recipient mice colonized with GM from young adult donors compared to those from old donors. This finding is in line with earlier studies showing that GF mice treated with SCFAs have increased muscle weight^30^ and conventionally raised mice treated with SCFAs are protected from muscle loss during aging.^35^

*B. ovatus* was the only bacterium identified at the species level, and was enriched in all recipient mice colonized with GM from young adult donors. *B. ovatus* has been proposed as a promising next-generation probiotic due to its preventive effects on lipopolysaccharides-associated inflammation^47^ and colitis in mice.^48–50^ *B. ovatus* can liberate N-methylserotonin from orange fibers, and GF mice fed a western diet and treated with N-methylserotonin had reduced adiposity and alterations in hepatic glucose metabolism.^51^ GF mice that were mono-colonized with *B. ovatus* produced propionate.^52^ Members of Bacteroides produce propionate through the succinate pathway.^53,54^ In line with these studies, recipient mice colonized with GM from young adult donors had increased levels of propionate in the present study. We have earlier observed that treatment of male orchiectomized mice and their controls with a probiotic mix increased lean mass and the levels of propionate in cecum.^55^ This is interesting but to our knowledge there are no studies demonstrating a direct effect of propionate on lean mass.

The relative abundance of *B. ovatus* was directly associated with the change in lean mass percentage in still growing recipient mice. In addition, *B. ovatus* was not present in any of the old donor mice with a low lean mass percentage, while it was present in all young adult donor mice with a high lean mass percentage. Furthermore, we observed that the presence of *B. ovatus* is associated with relatively lean mass in humans, using the large population-based HUNT study.^56,57^ Taken together, the presence of *B. ovatus* is associated with high relative lean mass in both mice and humans.

These findings underscore that additional preclinical studies to determine whether GMT from young mice can improve the musculoskeletal phenotypes of elderly frail recipient mice, and if so, what mechanistic role *B. ovatus* plays in mediating this effect.

## Materials and methods

### Donor mouse experiment

#### Housing of donor mice

Eighteen month-old and 2 month-old female C57BL/6J mice from Jackson Laboratory (Sacramento, Ca, USA) were housed for 3 months in a standard animal facility under controlled temperature (22°C), photoperiod (12 h light/dark cycle), with free access to fresh water, and a pelleted diet (LabDiet 5K52, Opend ApS, Denmark). At 21 months (old, *n* = 20) and 5 months (young adult, *n* = 10) of age, the mice were anesthetized with ketador/dexdomitor vet (Richter Pharma/Orion Pharma), bled from the axillary vein, and then euthanized by cervical dislocation. Tissues and cecal contents were snap-frozen in liquid nitrogen and maintained at −80°C. Bones were excised and fixed in 4% paraformaldehyde. All experimental procedures involving conventionally raised animals were approved by the regional animal ethics committee in Gothenburg.

### Dual-energy x-ray absorptiometry (DXA)

Lean mass percentage was analyzed prior to euthanasia using Faxitron UltraFocus dual-energy X-ray absorptiometry (Faxitron Bioptics, Tuscon, AZ, USA). The mice were scanned using an X-ray energy of 40 kV and 0.28 mA for 2.53 s with a spatial resolution of 24 µm using 2X geometric magnification. Images were analyzed using the VISION DXA software (Faxitron Bioptics, Tuscon, AZ, USA).

### Gnotobiotic mouse experiments

All experiments involving gnotobiotic mice were performed using protocols approved by the Washington University Institutional Animal Care and Use Committee (IACUC). Germ-free (GF) female C57BL/6J mice were housed in plastic flexible film isolators (Class Biologically Clean Ltd) at 23°C under a strict 12-h light cycle (lights on at 0600 h). The cages contained paper houses for environmental enrichment and autoclaved bedding (Aspen Woodchips; Northeastern Products).

### Selection and preparation of cecal contents for colonization

Four of the conventionally raised donor mice (two old and two young adults) were selected for GMT into recipient GF mice (*n* = 12/treatment group). Donor mice were selected from a larger group of 20 old and 10 young adult mice based on the representation of cecal gut microbial community members, as defined by sequencing amplicons generated by PCR from variable region 4 of bacterial 16S rRNA genes [see below for description of the methods used for V4-16S rRNA sequencing and the generation of amplicon sequence variants (ASVs)]. To this end, we performed Indicator Species Analysis to identify bacterial taxa most representative of an old or young adult microbial community configuration using the ‘indicspecies’ package.^58^ in R. A list of indicator species with significant p-values was assembled for both groups, and a heatmap of the relative abundance of these taxa was generated in all potential donor mice (Table S1 and Fig. S12). Hierarchical clustering revealed subgroups with similar community configurations of indicator taxa (‘pheatmap’ package in R) among the donors. Potential donors were selected for each subgroup.

Following donor selection, the previously frozen cecal sample of each selected donor mouse was weighed, thawed, and transferred to a Coy anaerobic chamber (atmosphere: 75% N2, 20% CO2, 5% H2). The cecal material (approximately 100 mg per donor) was resuspended in 4 mL of anaerobic PBS supplemented with 0.05% L-cysteine HCl and transferred to a 50 mL conical tube containing 5 mL of sterile glass beads. The material was vortexed at maximum speed for 2 min and then passed through a sterile 100 µm pore diameter filter. The filtrate was then combined with an equal volume of sterile anaerobic PBS containing 30% glycerol and 0.05% L-cysteine HCl. The solution was aliquoted into 1.8 mL glass tubes (E-Z vials, Wheaton) that were crimped with covers containing a PTFE/gray butyl liner (Wheaton) and then frozen at −80°C.

### Colonization and monitoring of gnotobiotic mice

GF C57BL/6J female mice were weaned at 3 weeks of age on an autoclaved mouse chow diet (B&K Universal, East Yorkshire, UK; diet 7,378,000). Three days before the start of each experiment, the mice were switched to a diet identical to that consumed by the original donor mice (LabDiet 5K52). Prior to colonization, the mice were distributed to four gnotobiotic isolators according to their body weight, such that each isolator was populated with mice with similar average weights. Mice were colonized with donor microbiota at 5, 11, or 17-weeks of age via a single oral gavage of 200 µL of the prepared mixture (*n* = 12/treatment group). The mice were monitored daily for the next week. Body composition was characterized 2 and 35 days after gavage using an EchoMRI 3 in1 instrument (EchoMRI; Houston, TX, USA). All mice were euthanized by cervical dislocation, without prior fasting, 35 days after colonization. Tissues and cecal contents were snap-frozen in liquid nitrogen and maintained at −80°C. Bones were excised and fixed in 4% paraformaldehyde.

### All mouse experiments

#### Bacterial V4 16S rRNA profiling of microbial communities

DNA was extracted from cecal contents by resuspending the samples (~10–50 mg) in a tube containing 500 µL of Buffer A (200 mM Tris pH 8, 200 mM NaCl, 20 mM EDTA), 210 µL 20% SDS, 500 µL phenol:chloroform:isoamyl alcohol (pH 7.9, 25:24:1, Invitrogen), and 500 µL 0.1 mm diameter zirconia/silica beads. A metal ball bearing was added to each extraction tube. Resuspended cecal contents were disrupted by bead beating at maximum speed for 4 min at room temperature (BioSpec Products, Bartlesville, OK, USA). The samples were centrifuged at 12,000 × g for 5 min at 4°C. The aqueous phase was removed, and DNA was purified (Qiaquick columns, Qiagen, Hilden, Germany), eluted in 70 µL Tris-EDTA (TE) buffer, and quantified (Quant-iT dsDNA broad-range kit; Invitrogen). The concentration of all DNA samples was adjusted to 1 ng/µL, and the DNA was subjected to PCR using barcoded primers directed against variable region 4 (V4) of the bacterial 16S rRNA gene. PCR was performed under the following cycling conditions: denaturation (94°C for 2 min) followed by 26 cycles of 94°C for 15 s, 50°C for 30 s, and 68°C for 30 s; and incubation at 68°C for 2 min. Amplicons were quantified prior to pooling and sequencing on an Illumina MiSeq instrument using paired-end 250 nucleotide reads. DNA sequences were demultiplexed, oriented, and trimmed to remove the primer sequences. DADA2 (1.10.1) was used to remove chimeric sequences and identify and quantify ASVs. Taxonomic assignments were made using the RDP Naive Bayesian Classifier algorithm and GreenGenes (13.8) training set.

### Bioinformatics

ASVs were used to generate multiple sequence alignments with MUSCLE^59^/3.8.31. Phylogenetic trees were generated using the FastTree^60^/2.1.11. The datasets with their phylogenetic trees were loaded into R^61^/4.0.1, and transformed into phyloseq objects using the phyloseq package^62^/1.32.0. Taxonomic counts were normalized by dividing the feature by the sum of counts for each sample, and the resulting fraction was multiplied by 100. Phyloseq was used to investigate beta diversity. UniFrac^63^ (weighted and unweighted) distance dissimilarity was calculated, and the resulting matrices were used in principal coordinate analysis (PCoA). The statistical significance of the difference in beta diversities was calculated using permutational ANOVA (PERMANOVA) within phyloseq. Beta dispersions were estimated and tested using permutation tests in the vegan.^64^ The alpha diversities were calculated with Chao1^65^, Simpson,^66^ and Shannon index^67^ within the phyloseq. LDA effect size^68^ (LefSE) was used to investigate differences in ASV representation between groups.

### Serum analysis

We used an ELISA kit to measure serum amyloid A (SAA) in serum (R&D, Systems, Minneapolis, MN, USA).

### High-resolution micro-computed tomography (µCT)

High-resolution µCT was used to analyze the tibia of donor mice (Skyscan 1172 scanner, Bruker MicroCT, Aartselaar, Belgium) and recipient mice (SkyScan 1275, Bruker MicroCT, Aartselaar, Belgium) as previously described.^69^ Tibiae of donor mice were imaged with an X-ray tube voltage of 50 kV, a current of 200 µA, and a 0.5 mm aluminum filter. The scanning angular rotation was 180° and the angular increment was 0.70°. The voxel size was 4.3 µm isotropically. NRecon (version 1.6.9) was used to perform reconstruction after the scans. The trabecular bone proximal to the distal tibial growth plate was selected for analyses within a conforming volume of interest (cortical bone excluded), starting 620 µm from the growth plate and extending 128 µm in the proximal direction. Cortical measurements were performed in the diaphyseal region of the tibia starting 4.99 mm from the growth plate and extending 128 µm in the proximal direction. Tibiae of recipient mice were imaged with an X-ray tube voltage of 40 kV, a current of 200 µA, and a 1 mm aluminum filter. The scanning angular rotation was 180° and the angular increment was 0.40°. The voxel size was 7 µm isotropically. The trabecular bone proximal to the distal tibial growth plate was selected for analyses within a conforming volume of interest (cortical bone excluded), starting 504 µm from the growth plate and extending 210 µm in the proximal direction. Cortical measurements were performed in the diaphyseal region of the tibia starting 5.25 mm from the growth plate and extending 210 µm in the proximal direction. The data were analyzed using CTAn software (Bruker MicroCT).

### Three-point bending of humerus

After dissection, the humerus was frozen at −20°C. Three-point bending was performed with a span length of 4.5 mm and loading speed of 0.155 mm/s using an Instron 3366 instrument (Instron, Norwood, MA, USA). Biomechanical parameters based on the recorded load deformation curves were calculated using Bluehill 2 software v.2.6 (Instron) with custom-made Excel (Microsoft) macros.

## Flow cytometry

Bone marrow cells from one femur per mouse were isolated and stained with eBioscience^TM^ Fixable Viability Dye eFluor^TM^ 780, according to the manufacturer’s protocol (Invitrogen, ThermoFisher Scientific). Cells were extracellularly stained with anti-CD3 (Clone 17A2, Nordic BioSite AB, Täby, Sweden), anti-CD4 (Clone RM4–5, Nordic BioSite AB, Täby, Sweden), and anti-CD25 (Clone PC61, BD, Franklin Lakes, NJ, USA). Cells were fixed and permeabilized using the FoxP3 staining buffer kit (Invitrogen, Thermofisher Scientific) and stained intracellularly with anti-Foxp3 (Clone FJK-16s, ThermoFisher Scientific) according to the manufacturer’s instructions. Treg cells were defined as CD4+CD25+Foxp3+. Samples from donor mice were run on a BD FACSVerse instrument (BD, Franklin Lakes, NJ, USA), whereas samples from recipient mice were analyzed using a BD FACSAria III cytometer (BD, Franklin Lakes, NJ, USA). Data were analyzed using FlowJo software (version 10; Tree Star, Inc.).

### Associations between *bacteroides ovatus* and lean mass in the HUNT study

Associations between *Bacteroides ovatus* (*B. ovatus*) and total and appendicular lean mass in humans were tested in a large population-based Norwegian study (the fourth Trøndelag Health Study; HUNT4).^56,57^ Participants were recruited between 2017 and 2019. In the present study, we used data from 4966 participants with data on *B. ovatus* measurements and total and appendicular lean mass. These participants were on average (± SD) 57.4 ± 14.3 years old (63.6% women) with an average (± SD) body weight of 78.0 ± 15.2 kg, total lean mass of 49.8 ± 10.0 kg, appendicular lean mass of 21.9 ± 4.7 kg. *B. ovatus* was measured in feces using quantitative PCR as previously described.^70^ Among the subjects with available fecal DNA that passed the quality criteria, *B. ovatus* was present in 79.6% of the subjects. The total body mass, appendicular lean mass, body fat mass, and height were determined using bioelectrical impedance analysis.^70^ Statistical analysis was performed in R. To assess the associations between the presence of *B. ovatus* and lean mass, we used linear regressions adjusted for age, sex, height, body fat mass, analysis batch, and batch-specific covariates. This study was approved by the Regional Committee for Medical and Health Research Ethics in Central Norway (reference number 2015/615). All the participants provided written informed consent.

### Statistical analyses

GraphPad prism (version 9.0.1) was used for statistical analyses. Results are presented as mean values ± SEM. Old and young adult donor mice were compared by Student’s t-test. Recipient mice colonized with GM from old or young adult donors were compared using two-way ANOVA to test the overall effect for the age of donor mice (old versus young adult), the age of recipient mice (GF mice), and the interaction effect. If the 2-way ANOVA showed an overall effect, we tested for differences between all groups using the Šídák post hoc test to determine if the effect was mainly in the 5-, 11- and/or 17-week-old recipient mice. Immune cells for recipient mice colonized with GM from old or young adult donors were statistically analyzed by Student’s t-test followed by Bonferroni correction (3 comparisons) since the samples from 5-, 11- and 17-week-old recipient mice were run on different occasions and cannot be compared due to interassay variation. *p* < .05 was considered significant.

## Supplementary Material

Supplemental Material

## Data Availability

Sequencing data are available from the NCBI database under the BioProject number PRJNA949823 in https://www.ncbi.nlm.nih.gov/bioproject/. Other data supporting the findings of this study are available from the corresponding authors upon reasonable request.
