# Supplementary material for: Transplantation of gut microbiota from old mice into young healthy mice reduces lean mass but not bone mass
Source: Gut Microbes. 2023 Jul 20;15(1):2236755. doi: 10.1080/19490976.2023.2236755 (PMC10364652; doi:10.1080/19490976.2023.2236755)
Supplement: Supplemental Material [file KGMI_A_2236755_SM1835.zip › Supplemental material/20230626 Supplemental figure legends.docx]

**Supplemental figure legends**

**Supplemental figure 1. Heatmap of taxa identified by indicator species analysis as discriminatory for old and young adult donor mice.** Cecal microbiota from 21-month-old-mice (old) and 5-month-old-mice (young adult) were analyzed by bacterial V4-16S rRNA amplicon sequencing and indicator species analysis. The taxa in the heatmap were identified as significant by indicator species analysis and shown as row-normalized relative abundances. Cecal microbiota from mice number 5 and 30 were used as “old donors” and from mice 6 and 17 were used as “young adult donors”.

**Supplemental figure 2. Gating strategy for CD4+ cells and regulatory T cells (Treg).** Cells were pre-gated for singlets and alive cells (negative for viability dye). Then we gated for CD3+ cells and from that population for CD4+ cells. Treg cells were gated as CD4+ cells positive for FOXP3 and CD25.

**Supplemental figure 3. Representative scatter plots of CD4+ cells in bone marrow.** Cecal microbiota from old or young adult donor mice was transplanted to GF mice of different ages. At the end of the experiment, bone marrow cells were harvested from femur and stained for CD4, Foxp3, and CD25. Representative images of CD4+ cell populations in bone marrow from flow cytometry analyses in donor mice (A), in recipient mice colonized at 5 weeks of age (B), in recipient mice colonized at 11 weeks of age (C) and in recipient mice colonized at 17 weeks of age (D).

**Supplemental figure 4. Representative scatter plots of Treg cells in bone marrow.** Cecal microbiota from old or young adult donor mice was transplanted to GF mice of different ages. At the end of the experiment, bone marrow cells were harvested from femur and stained for CD4, Foxp3, and CD25. Representative images of Treg populations in bone marrow from flow cytometry analyses in donor mice (A), in recipient mice colonized at 5 weeks of age (B), in recipient mice colonized at 11 weeks of age (C) and in recipient mice colonized at 17 weeks of age (D).

**Supplemental figure 5 The gut microbiota of recipient mice cluster depending on the age of donor mice.** Cecal microbiota from old or young adult donor mice was transplanted to GF mice at different ages. At the end of the study, cecal contents were analyzed by bacterial V4-16S rRNA amplicon sequencing and principal coordinate analysis (PCoA) based on weighted UniFrac distance for recipient mice colonized at 5 weeks of age (A), unweighted UniFrac distance for recipient mice colonized at 5 weeks of age (B), weighted UniFrac distance for recipient mice colonized at 11 weeks of age (C), unweighted UniFrac distance for recipient mice colonized at 11 weeks of age (D), weighted UniFrac distance for recipient mice colonized at 17 weeks of age (E), and unweighted UniFrac distance for recipient mice colonized at 17 weeks of age (F). Data were analyzed statistically by using PERMANOVA.

**Supplemental figure 6. The cecal microbiota of recipient gnotobiotic mice cluster with the gut microbiota of donor mice.** Cecal microbiota from old or young adult donor mice was transplanted to GF mice at different ages. At the end of the study, cecal contents were analyzed by bacterial V4-16S rRNA amplicon sequencing and principal coordinate analysis (PCoA) based on weighted UniFrac (A), unweighted UniFrac distance (B) for all recipient and donor mice, weighted UniFrac (C), unweighted UniFrac distance (D) for recipient mice colonized at 5 weeks of age and donor mice, weighted UniFrac distance (E), unweighted UniFrac distance (F) for recipient mice colonized at 11 weeks of age and donor mice, weighted UniFrac distance (G), and unweighted UniFrac distance (H) for recipient mice colonized at 17 weeks of age and donor mice. Arrows indicate the donor mice. Data were analyzed statistically by using PERMANOVA.

**Supplemental figure 7. Recipient mice transplanted with GM from old donors have altered relative abundances of bacterial phyla and lower alpha diversity indexes** **compared to GM from young adult donors.** Cecal microbiota from old or young adult donor mice was transplanted to GF mice of different ages. At the end of the experiment, cecal contents were collected and analyzed by bacterial V4-16S rRNA amplicon sequencing. The relative abundance of all phyla was analyzed for recipient mice colonized at 5 weeks of age (A), 11 weeks of age (B), and 17 weeks of age (C). Alpha diversity was determined by Chao1 (D), Shannon (E), and Simpson (F). The overall effect of age of donor mice (old vs young adult), age of recipient mice (GF mice), and their interaction were calculated using two-way ANOVA followed by Šídák post hoc test to correct for multiple comparisons. ***p≤0.001, ** p≤0.01 and *p≤0.05

**Supplemental figure 8. Gut microbiota from old donors reduces levels of propionate in recipient mice.**  Cecal microbiota from old or young adult donor mice was transplanted to GF mice at different ages. At the end of the study, cecal contents were collected and levels of short chain fatty acids were quantified by GC-MS; Acetate (A), Propionate (B), and Butyrate (C) and the intermediate metabolites to SCFA; Lactate (D) and Succinate (E). Values are given as mean ± SEM. The overall effect of age of donor mice (old vs young adult), age of recipient mice (GF mice) and their interaction were calculated using two-way ANOVA followed by Šídák post hoc test to correct for multiple comparisons. *p<0.05

**Supplemental figure 9. Serum amyloid A (SAA).** Cecal microbiota from old or young adult donor mice was transplanted to GF mice at different ages. SAA levels in serum were measured 35 days after colonization. Values are given as mean ± SEM. The overall effect of age of donor mice (old vs young adult), age of recipient mice (GF mice) and their interaction were calculated using two-way ANOVA followed by Šídák post hoc test to correct for multiple comparisons.

**Supplemental figure 10. Total body lean and fat mass.** Body composition was determined at the time of gavage and 5 weeks later using qMR to calculate difference (∆) in lean (A) and fat (B) mass. Values are given as the mean ± SEM. Data were analyzed by two-way ANOVA to analyze the overall effect of age of donor mice (old vs young adult), age of recipient GF mice, and their interaction followed by Šídák post hoc test to correct for multiple comparisons. **p<0.01

**Supplemental figure 11. The body weight and relative tissue weights.** Cecal microbiota from old or young adult donor mice was transplanted to GF mice at different ages. At the end of the study, mice were weighed (A). In addition, various tissues were dissected and weighed; M. quadriceps (B), Inguinal fat (C), Gonadal fat (D), Retroperitoneal fat (E), Uterus (F), and Liver (G). Tissue weights are presented as percentage of body weight. Values are given as mean ± SEM. The overall effect of age of donor mice (old vs young adult), age of recipient GF mice and their interaction were calculated using two-way ANOVA followed by Šídák post hoc test to correct for multiple comparisons.

**Supplemental figure 12. LEfSe of enriched taxa in relation to age of donors.** Cecal microbiota from old or young adult donor mice were transplanted to GF mice at different ages. The cecal contents of mice were analyzed by bacterial V4-16S rRNA amplicon sequencing and Linear Discriminant Analysis (LDA) Effect Size (LEfSe) (α ≤ 0.05 and LDA>2.0) to identify bacterial members with significant differences in their relative abundance between mice colonized with old or young adult GM at 5 weeks (A), and 11 weeks (B) of age. Arrows indicate *Bacteroides ovatus.*
