# Supplementary material for: Transplantation of gut microbiota from old mice into young healthy mice reduces lean mass but not bone mass
Source: Gut Microbes. 2023 Jul 20;15(1):2236755. doi: 10.1080/19490976.2023.2236755 (PMC10364652; doi:10.1080/19490976.2023.2236755)
Supplement: Supplemental Material [file KGMI_A_2236755_SM1835.zip › Supplemental material/20230626 Supplemental Tables.docx]

**Tables**

| Age of recipient mice | r-value | p-value |
| --- | --- | --- |
| 5 weeks | 0.62 | p=0.0016 |
| 11 weeks | 0.64 | p=0.0007 |
| 17 weeks | -0.08 | NS |

**Table 1. Correlation between relative abundance of *B. ovatus* and change in percentage of lean mass in mice.**

5-, 11- and 17-week-old GF mice received microbiota transplants from young adult or old donors and were euthanized 5 weeks later (n=12 mice/treatment group). The percentage of lean body mass was determined at the beginning and at the end of the experiment by qMR. At the end of the experiment, cecal contents were collected, the PCR products of variable region 4 of bacterial 16S rRNA genes were sequenced and the relative abundances of amplicon sequence variants (ASVs) were defined. Data were analyzed by Spearman correlation to determine the correlation coefficient (r-value) of the relative abundance of ASVs assigned to *B. ovatus* and Δlean%.
