# Supplementary figures and images for: Transplantation of gut microbiota from old mice into young healthy mice reduces lean mass but not bone mass
Source: Gut Microbes. 2023 Jul 20;15(1):2236755. doi: 10.1080/19490976.2023.2236755 (PMC10364652; doi:10.1080/19490976.2023.2236755)

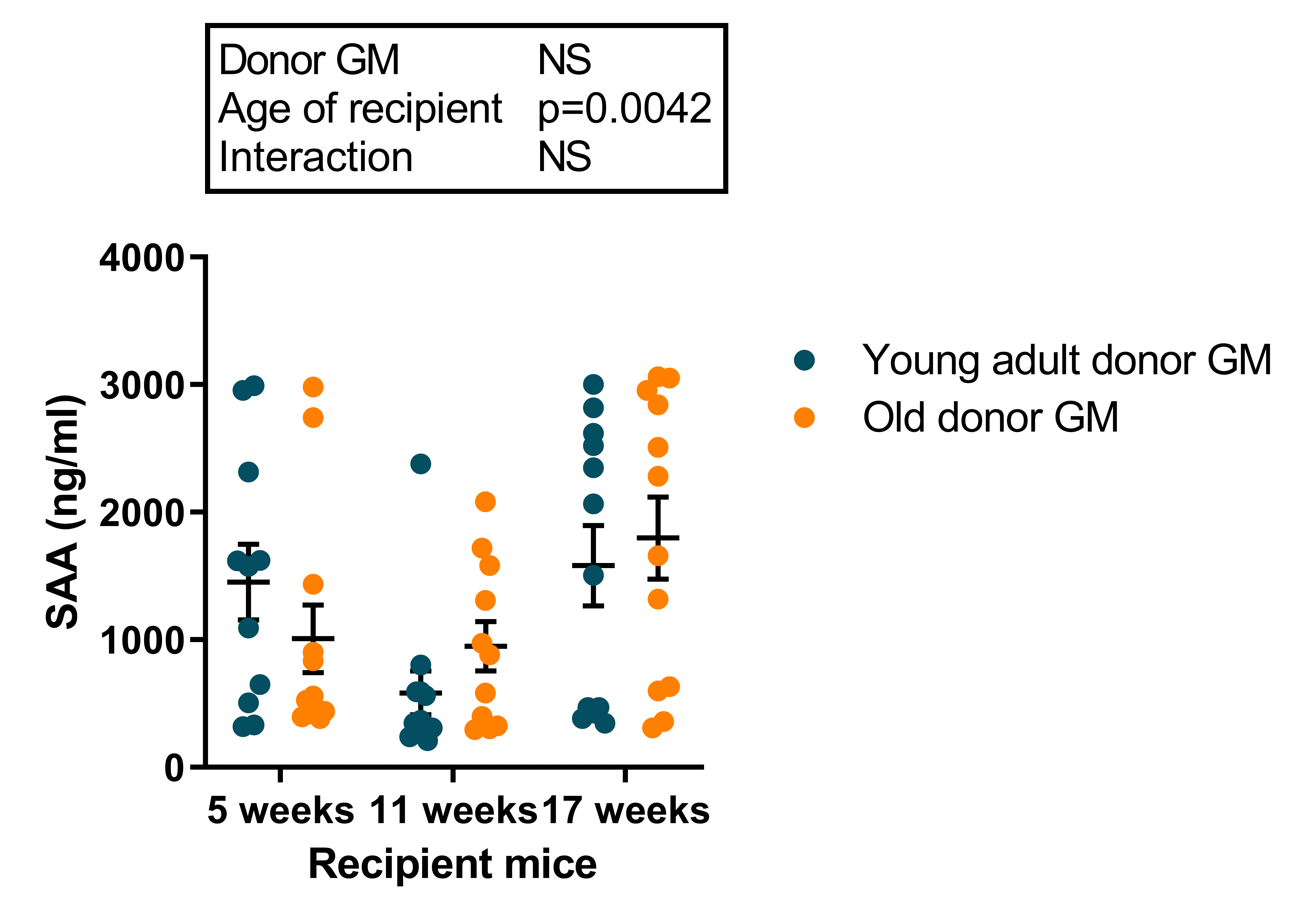

Supplement: Supplemental Material [file KGMI_A_2236755_SM1835.zip › Supplemental material/S Figure 8.tif]

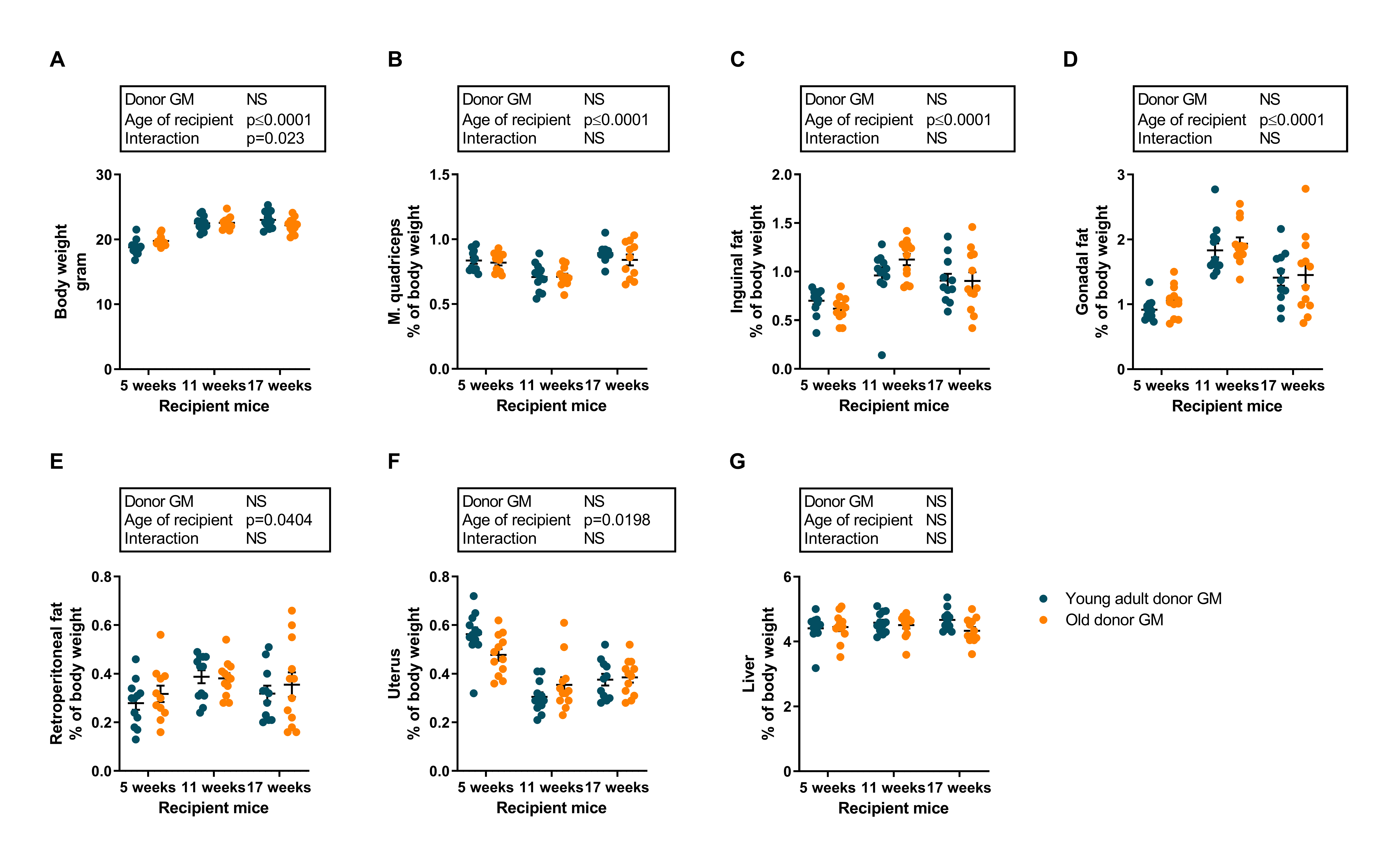

Supplement: Supplemental Material [file KGMI_A_2236755_SM1835.zip › Supplemental material/S. Figure 10.tif]

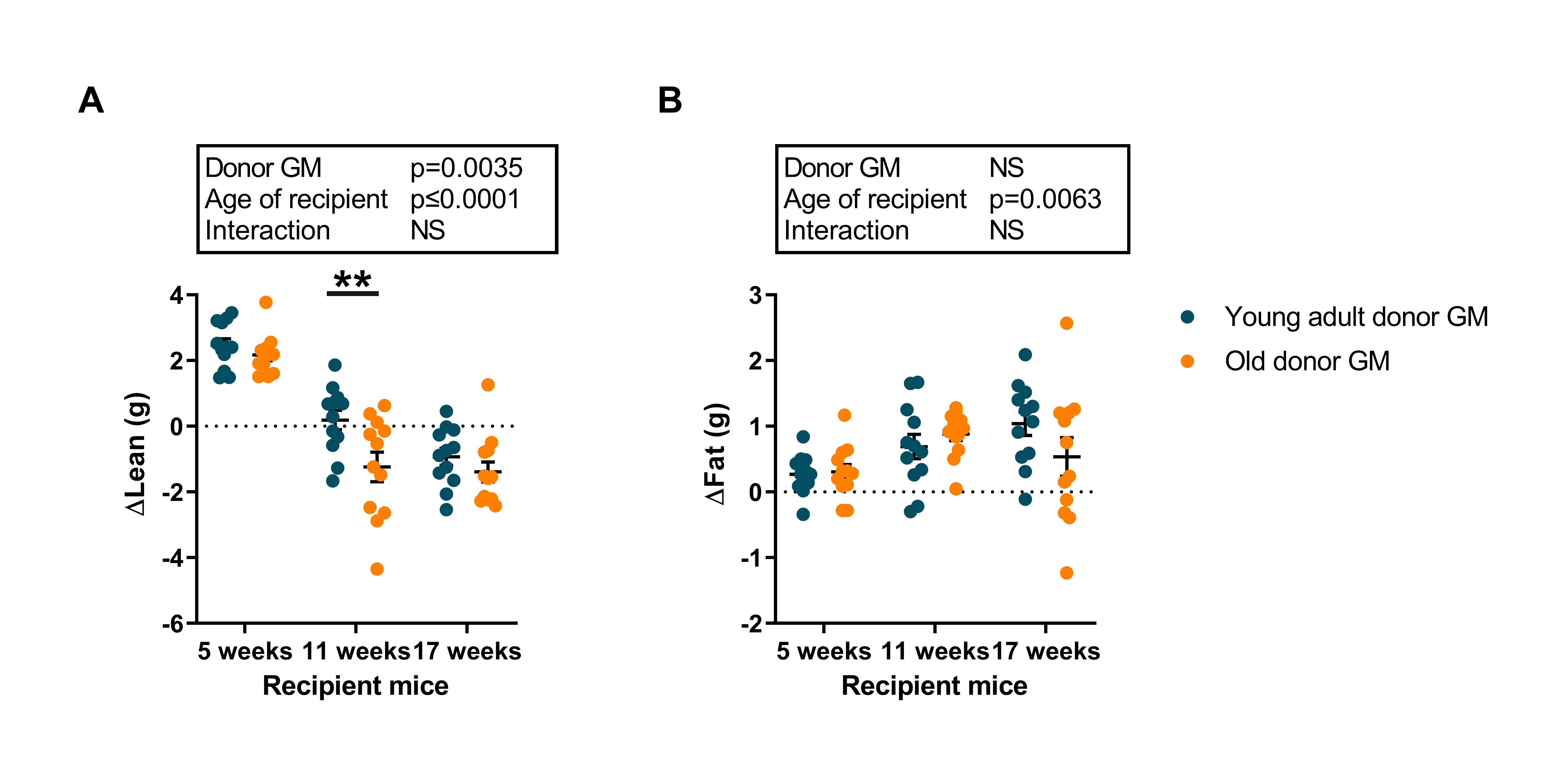

Supplement: Supplemental Material [file KGMI_A_2236755_SM1835.zip › Supplemental material/S. Figure 9.tif]
